# Nonstandard Finite Difference Method Applied to a Linear Pharmacokinetics Model

**DOI:** 10.3390/bioengineering4020040

**Published:** 2017-05-04

**Authors:** Oluwaseun Egbelowo, Charis Harley, Byron Jacobs

**Affiliations:** DST/NRF Centre of Excellence in the Mathematical and Statistical Sciences, School of Computer Science and Applied Mathematics, University of the Witwatersrand, Johannesburg, Private Bag 3, Wits 2050, South Africa; oluwaseun@aims.edu.gh (O.E.); Byron.Jacobs@wits.ac.za (B.J.)

**Keywords:** pharmacokinetic, standard finite difference, nonstandard finite difference, intravenous bolus injection, intravenous infusion

## Abstract

We extend the nonstandard finite difference method of solution to the study of pharmacokinetic–pharmacodynamic models. Pharmacokinetic (PK) models are commonly used to predict drug concentrations that drive controlled intravenous (I.V.) transfers (or infusion and oral transfers) while pharmacokinetic and pharmacodynamic (PD) interaction models are used to provide predictions of drug concentrations affecting the response of these clinical drugs. We structure a nonstandard finite difference (NSFD) scheme for the relevant system of equations which models this pharamcokinetic process. We compare the results obtained to standard methods. The scheme is dynamically consistent and reliable in replicating complex dynamic properties of the relevant continuous models for varying step sizes. This study provides assistance in understanding the long-term behavior of the drug in the system, and validation of the efficiency of the nonstandard finite difference scheme as the method of choice.

## 1. Introduction

The first attempt into what led to pharmacokinetics was described by Andrew Buchanan in his work *Physiological effects of the inhalation of ether* [1] in which he pointed out that for short ether inhalations, the speed of recovery of the ether was related to redistribution of ether in the body. Pharmacokinetics is the science of the kinetics of drug absorption, distribution, and elimination (more precisely relating to excretion and the metabolism). The mathematical representation of this work started with Michaelis and Menten [2] who first developed the now well-known Michaelis–Menten equation to describe enzyme kinetics; later this equation was also used to describe the elimination kinetics of drugs. Widmark and Tandberg [3] in 1924 published equations now known as (a) the one-compartment open model with bolus intravenous injection and multiple doses administered at uniform intervals and (b) the one-compartment open model with constant rate intravenous infusion [3]. Though the full concept of pharmacokinetics was introduced by Teorell [4], Holford and Sheiner [5] defined pharmacokinetics as a branch of pharmacology that employs mathematical models to better understand how drugs are absorbed, distributed, metabolized and excreted by the body. It has been well reported recently that the Food and Drug Administration (FDA) and other drug regulatory agencies have been using modeling and simulation to assist in making informed decisions. Furthermore, pharmaceutical companies are expected to justify their dose, their choice of patient population, and their dosing regimen, not just through clinical trials, but also through modeling and simulation [6,7].

After a drug is released from its dosage form, the drug is absorbed into the surrounding tissue, the body, or both. As commented on by Shargel et al. [8], the distribution through and elimination of the drug in the body varies for each patient but can be characterized using mathematical models and statistics. Being able to characterize drug distribution and elimination is an important prerequisite to be able to determine or modify the dosing regimens of individuals and groups of patients. From among the three main types of pharmacokinetic (PK) models—compartment, physiologic and statistical moment approach models—compartmentally-based models are known to be a very simple and useful tool in pharmacokinetics [8]. In essence, a compartment model provides a simple way of grouping all the tissues into one or more compartments where drugs move to and from the central or plasma compartment. Assuming a drug is given by intravenous (I.V.) injection and that the drug dissolves (distributes) rapidly in the body fluids, one may employ a one-compartment PK model that can describe the situation as a tank containing a volume of fluid that is rapidly equilibrated with the drug [8]. The concentration of the drug in the tank after a given dose is governed by two parameters, namely the fluid volume of the tank that will dilute the drug, and the elimination rate of the drug per unit of time—these are both assumed constant for a given drug [8]. Simplistic as this model may be with regards to drug distribution and elimination in the human body, a drug’s PK properties can frequently be described via such a one-compartment open model. Assuming such a model, the drug is both added to and eliminated from a central compartment which represents plasma and highly-perfused tissues that rapidly equilibrate with the drug. When an I.V. dose of drug is given, the drug enters directly into the central compartment while elimination of the drug occurs from the central compartment given that the organs involved in drug elimination, primarily the kidney and liver, are well-perfused tissues.

In a two-compartment model, the drug can move between the central or plasma compartment to and from the peripheral or tissue compartment [8]. Although the peripheral compartment does not represent a specific tissue, the mass balance accounts for the drug present in all the tissues. Knowing the parameters of either the one- or two-compartment model, one can estimate the amount of drug left in the body and the amount of drug eliminated from the body at any time. A drug that follows the pharmacokinetics of a two-compartment model does not equilibrate rapidly throughout the body, as is assumed for a one-compartment model [9]. In the former, the drug distributes into two compartments, the central compartment and the peripheral compartment. The central compartment represents the blood, extracellular fluid, and highly-perfused tissues. The drug distributes rapidly and uniformly in the central compartment. The peripheral compartment contains tissues in which the drug equilibrates more slowly. Drug transfer between the two compartments is assumed to take place by first-order processes. We will be considering this model in our work.

The simplicity and flexibility of the compartment model is the principal reason for its wide application. A major advantage of such models is that the time course of the drug in the body may be monitored quantitatively with a limited amount of data [10]. Furthermore, these models account accurately for the mass balance of the drug in the body and the amount of drug eliminated (Mass balance includes the drug in the plasma, the drug in the tissue pool, and the amount of drug eliminated after dosage administration.). Compartment models have successfully been applied for the prediction of the pharmacokinetics of the drug and the development of dosage regimens. Moreover, compartment models are very useful in relating plasma drug levels to pharmacodynamic (PD) and toxic effects in the body [10]. Underlying physiologic mechanisms can also be obtained via such a model through model testing of the data. Thus, compartment analysis may lead to a more accurate description of the underlying physiologic processes and the kinetics involved. In clinical PK literature, drug data comparisons are based on compartment models and the easy tabulation of important parameters accomplished [10].

In practice, PK models seldom consider all the rate processes ongoing in the body [9]. Due to the complexity models which incorporate such information pose, simplifying assumptions are often made so that solutions may be obtained. Traditional PK models, being simplified mathematical expressions, are based on the assumption of a linear relationship between the dose of a drug and its concentration [11]. In a linear model, these rate coefficients, called *k*, are assumed to be constant. However, such assumptions regarding the linearity of the model do not necessarily describe the actual physical processes as accurately as a non-linear relationship may. In fact, the non-linearities seen in such models are related to drug absorption, distribution, metabolism and excretion, and the pharmacokinetics of drug action. Another example where simplifying assumptions are made pertains to the number of tissue compartments in a perfusion model. Multi-compartment models were developed to explain the observation that, after a rapid I.V. injection, the plasma level–time curve does not decline linearly as a single, first-order rate process [9]. The plasma level–time curve reflects first-order elimination of the drug from the body only after distribution equilibrium, or plasma drug equilibrium with peripheral tissues occurs. Therefore, while the number of tissue compartments in a perfusion model does vary with the drug, the tissues or organs that have no drug penetration are invariably excluded from consideration. Organs such as the brain, the bones, and other parts of the central nervous system are often excluded, as most drugs have little penetration into these organs [8]. To describe each organ separately with a differential equation would make the model very complex and mathematically difficult to solve. Under such circumstances more sophisticated methods of solution need to be employed.

Our research is aimed at the well-known two-compartment model. We have chosen to consider the two-compartment model in this study, given that the one-compartment model assumes immediate distribution of the drug and attainment of equilibrium throughout the body. Realistically however, very few drugs display these characteristics, and hence we turn to a two-compartment model for consideration. While still a simplification of the actual physics of the problem, it is a well-justified model as commented on above. This model is capable of providing data on rates in and out of specific organs, which is of interest. The work conducted here is done with the aim of introducing a numerical method which may be employed in future research for the solution of models which are non-linear and describe multiple compartments. As such, we propose and illustrate the use of a numerical method of solution, namely the nonstandard finite difference method (NSFD), capable of efficiently obtaining solutions which are not only accurate but maintain the underlying dynamics of the system of equations. This choice of method impacts on whether we are able to consider non-compartment models; the NSFD is not amenable to the simulation of non-compartment models as it provides a meta-analysis of the inter-compartment dynamics, whereas non-compartment models are unable to describe these meta-dynamics and instead conduct parameter estimation of the entire system as a whole through the use of experimental data. The advantage of the NSFD method is the ability to predict the concentration–time profile of a drug when there are alterations in the dosing regimen—this would not be possible were one to consider non-compartment analysis. Another advantage of the NSFD method is that it preserves significant properties of the analogous models and consequently gives reliable numerical results even when analytical solutions are not possible. The standard approaches to multi-compartment models assume linear dynamics over the duration of each time step, whereas the NSFD method assumes exponential dynamics. Thus, in the case of a linear model the NSFD method recovers the model dynamics exactly. This paper illustrates the ability of the NSFD method to solve a two-compartment PK model in a stable and robust fashion, with the ability of being extended to non-linear and/or multi-compartment models.

## 2. Methods

### 2.1. Finite Difference Method

While the implementation of the NSFD method is the focus of this research, we employ the standard finite difference (SFD) method as a means of comparison. We define finite difference methods as numerical methods used for the solution of differential equations by approximating them with difference equations, in which finite differences approximate the derivatives.

We will introduce the concept of the SFD method from Taylor’s theorem, where *h* is termed the step size between the values of the independent variable *x*. Were we to increase *x* by *h* then according to Taylor’s theorem we could create a Taylor series expansion:

$$f\left(x+h\right)=f\left(x\right)+hf^{\prime }\left(x\right)+\frac{h^{2}}{2!}f^{\prime \prime }\left(x\right)\cdots$$

such that,

$$f^{\prime }\left(x\right)=\frac{f(x+h)-f(x)}{h}+O\left(h\right).$$

Under the assumption that h→0 then,

(1) $$f^{\prime }\left(x\right)\approx \frac{f(x+h)-f(x)}{h}\;\mathrm{or}\;f^{\prime }\left(x_{n}\right)\approx \frac{f_{n+1}-f_{n}}{h}.$$

Similarly, if we expand f(x) at f(x−h) then,

$$f\left(x-h\right)=f\left(x\right)-hf^{\prime }\left(x\right)+\frac{h^{2}}{2!}f^{\prime \prime }\left(x\right)\cdots$$

such that,

$$f^{\prime }\left(x\right)=\frac{f(x)-f(x-h)}{h}+O\left(h\right),$$

which, if h→0, gives,

(2) $$f^{\prime }\left(x\right)=\frac{f(x)-f(x-h)}{h}\;\mathrm{or}\;f^{\prime }\left(x_{n}\right)\approx \frac{f_{n}-f_{n-1}}{h}.$$

Subtracting the Taylor series expansion of f(x−h) from f(x+h) provides us with:

$$f\left(x+h\right)-f\left(x-h\right)=2hf^{\prime }\left(x\right)+\frac{2h^{3}}{3!}f^{\prime \prime \prime }\left(x\right)+\cdots$$

which, under the assumption that h→0, provides us with a third approximation for the derivative:

(3) $$f^{\prime }\left(x\right)=\frac{f(x+h)-f(x-h)}{2h}\;\mathrm{or}\;f^{\prime }\left(x_{n}\right)\approx \frac{f_{n+1}-f_{n-1}}{2h}.$$

Equations (1)–(3) are known as forward difference, backward difference and central difference approximations to the first order derivative, respectively. the approximation given by Equation (3) has an error of $O(h^{2})$ and is hence deemed the most accurate of the three approximations provided.

### 2.2. Nonstandard Finite Difference Method

The NSFD schemes developed by Mickens et al. [12,13,14,15,16] were proposed to compensate for the weaknesses of methods such as the SFD methods; numerical instabilities being a prime example. As commented on by Liao and Ding [17], with regard to the positivity, boundedness, and monotonicity of solutions, NSFD schemes have performed better than SFD schemes. Because it is more flexible in its construction, an NSFD scheme can more easily preserve certain properties and structures obeyed by the original equations and can have better dynamical consistency for dynamical problems [17]. The advantages of NSFD methods have been observed when being employed for many numerical applications. González-Parra et al. [18,19] developed some NSFD methods to preserve the positivity condition and population conservation law of biological models. Heat transfer problems have also been considered via this method in Jordan [20] and Malek [21].

The initial foundation of NSFD schemes came from exact finite difference schemes [22]. It is thought that numerical methods that approximate differential systems are expected to be consistent with the original differential systems. Mickens [23] provides a theorem which states that each ordinary differential equation corresponds to an “exact” finite difference scheme. He also introduces the NSFD method for designing schemes that are dynamically consistent with the original differential systems, preserve physical properties, obtain reliable results and require less effort to implement than those obtained via standard methods [24]. In this study, we employ the NSFD method to obtain solutions for the two-compartment I.V. bolus injection and the two-compartment I.V. infusion models. We furthermore, conduct a comparative analysis by also considering an analytical solution, the SFD method and ODE45 in MATLAB (MathWorks, Natick, MA, USA). The purpose of this paper is to obtain an “exact” finite difference scheme for a linear PK model using the procedure of Mickens [14]. In this fashion we hope to prove the degree to which this method may provide meaningful solutions to equations within this context.

#### NSFD Modeling Fundamental Principles

NSFD methods provide numerical solutions to differential equations by constructing discrete models. They preserve the significant properties of their continuous analogues and consequently give reliable numerical results. The following rules were given by Mickens in [25] for constructing an NSFD scheme:

**Rule** **1**. The orders of the discrete representation of the derivative must be equal to the orders of the corresponding derivatives appearing in the differential equations.

**Remark** **1.** If the order of the discrete representations for derivatives are larger than those occurring in the differential equations, then numerical instabilities will occur.

**Rule** **2**. Denominator functions for the discrete representations for derivatives must, in general, be expressed in terms of more complicated functions of the step-sizes than those conventionally used.

**Remark** **2.** *Consider a first-order differential equation of the form:*

(4) $$\begin{array}{c} \frac{du}{dt}=f\left(t,u,\lambda \right). \end{array}$$

*The conventional denominator* Δt *of the system in (4) is:*

(5) $$\begin{array}{c} \frac{du}{dt}\to \frac{u_{k+1}-u_{k}}{\Delta t}, \end{array}$$

*which is replaced by a nonnegative function* ϕ(Δt) *where,*

(6) $$\begin{array}{c} \phi \left(h\right)=h+O\left(h^{2}\right). \end{array}$$

*In the above, we have:*

(7) $$\begin{array}{c} h=\Delta t,\;t\to t_{k}=hk,\;u\left(t\right)\to u_{k}, \end{array}$$

*where* k *is an integer. The exact discrete representations of Equation (4) are given as:*

(8) $$\begin{array}{c} \frac{du}{dt}\to \frac{u_{k+1}-\varphi u_{k}}{\phi (h)}, \end{array}$$

*where,*

φ(h)=1+O(h).

**Rule** **3**. Nonlinear terms must, in general, be modeled by nonlocal discrete representations.

**Remark** **3.** *The nonlinear terms that occur in* f(t,u,λ) *are approximated in a nonlocal way by a suitable function of several points on the mesh. For example, Mickens’ approximation of nonlinear terms [14,26,27] is given as:*

(9) $$\begin{array}{c} u^{2}\approx u_{k}u_{k+1}\;and\;u^{3}\approx \frac{2u_{k+1}^{2}u_{k}^{2}}{u_{k+1}+u_{k}}, \end{array}$$

*Erdogan and Ozis’ approximation of the nonlinear term, which was clearly stated in [27], is given as:*

(10) $$\begin{array}{c} u^{2}\approx u_{k}u_{k+1}\;and\;u^{3}\approx \frac{1}{2}u_{k}^{2}\left(3u_{k+1}-u_{k}\right). \end{array}$$

**Rule** **4**. All the special conditions that correspond to either the differential equation and/or its solutions should also correspond to the difference equation and/or its solutions.

**Remark** **4.** *For example, the system described in Equation (8) is called time-invariant if the behavior of the system does not explicitly depend on the absolute time. In other words, if* $f\left(t_{1},u,\lambda \right)=f\left(t_{2},u,\lambda \right)$ *for any two times* $t_{1}$ *and* $t_{2},$ *then the system is time-invariant. If the discrete representation of the same model does not also have this property, numerical instabilities may occur.*

**Rule** **5**. The discrete scheme should not introduce extraneous or spurious solutions.

**Remark** **5.** The discrete representation of the derivative must, in general, converge to the same fixed-point solutions as the corresponding derivative. If it does otherwise, numerical instabilities may occur.

### 2.3. Phase Plane Analysis

For a system of linear differential equations ${\underline{x}}^{\prime }=A\underline{x}$, the phase portrait is a representative set of its solutions, plotted as parametric curves (with *t* as the parameter) on the Cartesian plane tracing the path of each particular solution $\left(x,y\right)=(x_{1}\left(t\right),x_{2}\left(t\right))$ where 0<t<∞. Thus, by evaluating $A\underline{x}$ at a large number of points and plotting the resulting vectors, one obtains a direction field of tangent vectors to solutions of the system of differential equations. This phase portrait is a graphical tool which assists us in visualizing how the solutions of a given system of differential equations would behave as time evolves. In this context, the Cartesian plane where the phase portrait resides is called the phase plane. The parametric curves traced by the solutions are sometimes also called their trajectories. A qualitative understanding of the behavior of the solutions and local stability of the numerical solution near steady states can usually be gained from a direction field. More precise information can be discovered by including in the plot some solution curves or trajectories.

A phase portrait is able to assist us in establishing whether the trajectories of a solution will approach the equilibrium solution as *t* increases, where the equilibrium solution is obtained when $A\underline{x}=\underline{0}$. The behavior of these trajectories around equilibrium points provides further insight into the dynamics of the solution, particularly pertaining to varied parameter values.

## 3. Results

### 3.1. Compartmental Models for Pharmacokinetics

PK models deal with the mathematical description of the rates of the drug movement into, within and upon exiting the body. In this work we depict the whole system as two compartments; employing a two-compartment model is often more appropriate mathematically and physiologically when modeling the rate of a drug in the body. These models are used to predict the time course of drugs in the body and to allow maintenance of drug concentration in the therapeutic range by predicting drug levels in each compartment. In this work we will assume that the drug is uniformly distributed within each compartment. The compartments are also considered to be well-stirred and mixing of the drug is assumed to be rapid. Elimination is depicted as occurring in the central compartment, so the drug in the peripheral compartment must transfer back to the central compartment.

For the *q*-compartment, we have:

(11) $$\begin{array}{c} \frac{dc_{i}}{dt}=\sum_{j=0}^{q}\left(k_{ji}c_{j}-k_{ij}c_{i}\right), \end{array}$$

where $c_{i}\left(t\right)$ is the concentration of the drug in compartment *i*, i=1,2,…,q and the rate constant $k_{ij}$ governs the rate of the drug movement from the *i*th compartment to the *j*th-compartment and vice versa.

The variables of importance in this problem structure are as follows:

- *c* : Concentration of drug in central compartment.
- *p* : Concentration of drug in peripheral compartment.
- $k_{12}$: Transfer rate of drug from central to peripheral compartment.
- $k_{21}$: Degradation rate of drug in peripheral compartment.
- $k_{10}$: Clearance rate of drug leaving the central compartment.

The data relating to the pharmacokinetics of sisomicin, a new single component aminoglycoside antibiotic, were obtained from Péchère et al. [28] to test the model in this study. The elimination profile of this antibiotic follows two-compartment model kinetics after I.V. administration. The I.V. bolus injection is structured as the initial condition which has the units of concentration as mg/mL. In turn, the I.V. bolus infusion is administered as a dose, *D*, with unit mg/min, as shown in Table 1. The equations considered below are focused on obtaining the concentration in each compartment such that $C_{p}=\frac{A_{p}}{V_{p}}$ where $A_{p}$ is the amount of drug present in the $p^{th}$ compartment, $V_{p}$ is the volume of the drug in the $p^{th}$ compartment, with $C_{p}$ representing the compartments: *c* (central) and *p* (peripheral) [9]. As such, we consider the infusion *I* to be defined as $\frac{D}{V_{p}}$ with the unit $\frac{mg/min}{ml}$ [28].

**Figure 1:**
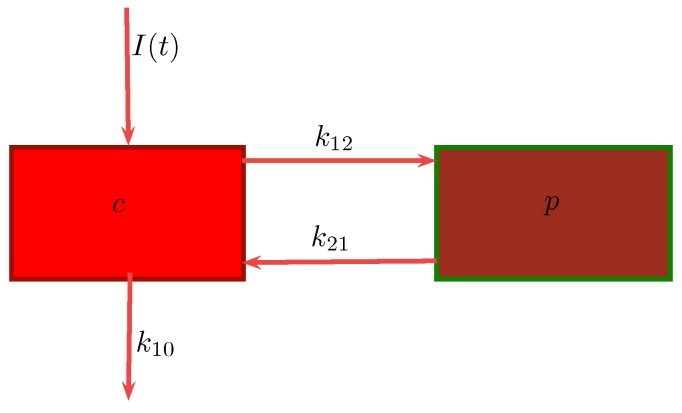
Two-compartment model with intravenous (I.V.) infusion introduced into the plasma compartment.

Suppose we consider a two-compartment I.V. infusion model. We employ c(t) and p(t) to represent the drug in the central and peripheral compartment, respectively, within the two-compartment model as depicted in Figure 1. The central compartment is identified with the blood while the peripheral compartment describes soft tissue. The absorption phase is omitted because the drug was administered with I.V. infusion at time zero. Then, Equation (11) reads:

(12) $$\begin{array}{c} \left\{\begin{array}{cc} \frac{dc}{dt} & =k_{21}p-k_{12}c-k_{10}c+I\left(t\right),\\ \frac{dp}{dt} & =k_{12}c-k_{21}p. \end{array}\right. \end{array}$$

We will provide both analytical and numerical solutions to this equation for the case: I(t)=0 and $I\left(t\right)=I_{0}$.

#### **Case 1**: I(t)=0

If I(t)=0 then (12) becomes a two-compartment I.V. bolus injection model, given as:

(13) $$\begin{array}{c} \left\{\begin{array}{cc} \frac{dc}{dt} & =k_{21}p-k_{12}c-k_{10}c,\;c\left(0\right)=1,\\ \frac{dp}{dt} & =k_{12}c-k_{21}p\;\;\;\;p\left(0\right)=0. \end{array}\right. \end{array}$$

#### **Case 2**: $I\left(t\right)=I_{0}$

If $I\left(t\right)=I_{0}$ then (12) becomes a two-compartment I.V. infusion model, given as:

(14) $$\begin{array}{c} \left\{\begin{array}{cc} \frac{dc}{dt} & =k_{21}p-k_{12}c-k_{10}c+I_{0},\;c\left(0\right)=0,\\ \frac{dp}{dt} & =k_{12}c-k_{21}p\;\;\;\;\;\;p\left(0\right)=0. \end{array}\right. \end{array}$$

The values considered are obtained from the work by Péchère et al. [28]. In this work sisomicin kinetic parameters were derived from a two-compartment open model analysis of serum data after I.V. administration. The values for Subjects 1–4 are employed for the dynamical systems analyses; for the numerical solutions obtained we considered the values of Subject 1. We have also considered different values of the dose; realistically these alternate values should correspond to different values of $I_{0}$, however the assumption is that the parameter values are within the correct range to provide us with at least a semblance of the correct dynamics.

##### 3.2. Analytical Solution

###### 3.2.1. Case 1

We obtain the analytical solution for the blood compartment c(t) and tissue compartment p(t) to Equation (13) via the Laplace transform L. The Laplace transform is an integral transformation where the linear operator L{f(t)} transforms a function f(t) with $t\in R_{\geq 0}$ from the time domain to a function f(s) with s∈C in an image domain [29,30]. The main advantage of the Laplace transform is that differentiation and integration in the time domain corresponds to simple algebraic operations in the image domain. Transforming Equation (13) and writing $Q_{1}$, $Q_{2}$ for L{c} and L{p} respectively, we obtain:

(15) $$\begin{array}{c} \left\{\begin{array}{c} \left(s+k_{10}+k_{12}\right)Q_{1}-k_{21}Q_{2}=1\\ -k_{12}Q_{1}+\left(s+k_{21}\right)Q_{2}=0, \end{array}\right. \end{array}$$

upon substitution of the initial conditions and rearranging accordingly. These are simultaneous algebraic equations in $Q_{1}$ and $Q_{2}$; we can solve for these upon employing the method of elimination:

$$\begin{array}{ccc} Q_{2}\left(-s^{2}-sk_{21}-sk_{10}-k_{10}k_{21}-sk_{12}-k_{12}k_{21}+k_{12}k_{21}\right) & = & -k_{12}\\ \left(s^{2}+\left(k_{10}+k_{12}+k_{21}\right)s+k_{10}k_{12}\right)Q_{2} & = & k_{12}\\ Q_{2} & = & \frac{k_{12}}{s^{2}+\left(k_{10}+k_{12}+k_{21}\right)s+k_{10}k_{12}} \end{array}$$

providing,

(16) $$\begin{array}{ccc} Q_{2} & = & \frac{k_{12}}{\left(s+\lambda_{1}\right)\left(s+\lambda_{2}\right)} \end{array}$$

where,

(17) $$\lambda_{1,2}=\frac{1}{2}\left(k_{10}+k_{12}+k_{21}\pm \sqrt{{\left(k_{10}+k_{12}+k_{21}\right)}^{2}-4k_{10}k_{21}}\right)$$

such that,

$$\begin{array}{c} \lambda_{1}+\lambda_{2}=k_{12}+k_{21}+k_{10},\;\lambda_{1}\lambda_{2}=k_{21}k_{10}. \end{array}$$

To obtain the solution for the peripheral compartment we take inverse Laplace transform of Equation (16) which gives:

(18) $$\begin{array}{ccc} L^{-1}\left\{Q_{2}\right\}=p\left(t\right) & = & L^{-1}\left\{\frac{k_{12}}{\left(s+\lambda_{1}\right)\left(s+\lambda_{2}\right)}\right\}\\ & = & k_{12}L^{-1}\left\{\frac{A}{s+\lambda_{1}}+\frac{B}{\left(s+\lambda_{2}\right)}\right\}, \end{array}$$

where,

$$\begin{array}{c} A=\frac{k_{12}}{\lambda_{2}-\lambda_{1}},\;B=\frac{-k_{12}}{\lambda_{2}-\lambda_{1}}\; \end{array}$$

such that,

$$\begin{array}{ccc} p(t) & = & \frac{k_{12}}{\lambda_{2}-\lambda_{1}}\left(\exp\left(-\lambda_{1}t\right)-\exp\left(-\lambda_{2}t\right)\right). \end{array}$$

This then allows us to find c(t) by eliminating $Q_{2}$ in Equation (15) or by substituting the solution for p(t) in one of the equations given in (13). In this case, it will be simpler to substitute for p(t) in the second equation of (13), giving,

$$\begin{array}{ccc} k_{12}c & = & \frac{dp}{dt}+k_{21}p\left(t\right)\\ & = & \frac{d}{dt}\left(\frac{k_{12}}{\lambda_{2}-\lambda_{1}}\left(\exp\left(-\lambda_{1}t\right)-\exp\left(-\lambda_{2}t\right)\right)\right)+k_{21}\left(\frac{k_{12}}{\lambda_{2}-\lambda_{1}}\left(\exp\left(-\lambda_{1}t\right)-\exp\left(-\lambda_{2}t\right)\right)\right)\\ & = & \frac{k_{12}}{\lambda_{2}-\lambda_{1}}\left(-\lambda_{1}\exp\left(-\lambda_{1}t\right)+\lambda_{2}\exp\left(-\lambda_{2}t\right)\right)+\frac{k_{12}k_{21}}{\lambda_{2}-\lambda_{1}}\left(\exp\left(-\lambda_{1}t\right)-\exp\left(-\lambda_{2}t\right)\right) \end{array}$$

such that,

$$\begin{array}{ccc} c(t) & = & \frac{\lambda_{1}-k_{21}}{\lambda_{1}-\lambda_{2}}\exp\left(-\lambda_{1}t\right)+\frac{k_{21}-\lambda_{2}}{\lambda_{1}-\lambda_{2}}\exp\left(-\lambda_{2}t\right). \end{array}$$

The concentration in the central compartment is hence,

(19) $$\begin{array}{c} c\left(t\right)=E\exp\left(-\lambda_{1}t\right)+\left(1-E\right)\exp\left(-\lambda_{2}t\right) \end{array}$$

and the concentration of the drug in the peripheral compartment is:

(20) $$\begin{array}{c} p\left(t\right)=\frac{k_{12}}{\lambda_{2}-\lambda_{1}}\left(\exp\left(-\lambda_{1}t\right)-\exp\left(-\lambda_{2}t\right)\right), \end{array}$$

where $E=\frac{\lambda_{1}-k_{21}}{\lambda_{1}-\lambda_{2}}.$

###### 3.2.2. Case 2

The general solution for (14) is of the form $q\left(t\right)=q_{p}+q_{c}$ where $q_{p}$ is a particular solution and $q_{c}$ is the complementary solution, i.e., the general solution of the homogeneous part of the Equation (14). To obtain a particular solution of Equation (14), we assume c(t) and p(t) to be constant, such that,

(21) $$k_{21}p-k_{12}c-k_{10}c+I_{0}=0$$

and,

(22) $$k_{12}c-k_{21}p=0.$$

Upon substituting (21) into (22) we obtain the particular solution of (14):

(23) $$q_{p}=\left(\begin{array}{c} c(t)\\ p(t) \end{array}\right)=\frac{I_{0}}{k_{10}}\left(\begin{array}{c} 1\\ \frac{k_{12}}{k_{21}} \end{array}\right).$$

To find the complementary solution to Equation (14) at I(t)=0) we consider,

(24) $$\left(\begin{array}{c} c^{\prime }\\ p^{\prime } \end{array}\right)=\left(\begin{array}{cc} -k_{10}-k_{12} & k_{21}\\ k_{12} & -k_{21} \end{array}\right)\left(\begin{array}{c} c\\ p \end{array}\right)$$

providing the corresponding matrix,

(25) $$A=\left(\begin{array}{cc} -k_{10}-k_{12} & k_{21}\\ k_{12} & -k_{21} \end{array}\right).$$

We can find a fundamental set of solutions for the matrix by finding the eigenvalues and the corresponding eigenvectors. The matrix *A* has eigenvalues λ such that:

(26) $$\lambda^{2}+\left(k_{10}+k_{12}+k_{21}\right)\lambda +k_{10}k_{21}=0$$

which, upon solution, provides the following eigenvalues:

(27) $$\lambda_{1,2}=\frac{1}{2}\left(-k_{10}-k_{12}-k_{21}\pm \sqrt{\left(k_{10}+k_{12}+k_{21}\right){}^{2}-4k_{10}k_{21}}\right).$$

Suppose $\underline{v}=\left(\begin{array}{c} v_{1}\\ v_{2} \end{array}\right)$ are the eigenvectors corresponding to the eigenvalues, then $\left(A-\lambda I\right)\underline{v}=0$. Hence we have that,

$$\left(\begin{array}{cc} -k_{10}-k_{12}-\lambda_{1,2} & k_{21}\\ k_{12} & -k_{21}-\lambda_{1,2} \end{array}\right)\left(\begin{array}{c} v_{1}\\ v_{2} \end{array}\right)=\left(\begin{array}{c} 0\\ 0 \end{array}\right)$$

or,

(28) $$\begin{array}{c} \left\{\begin{array}{c} \left(-k_{10}-k_{12}-\lambda_{1,2}\right)v_{1}+k_{21}v_{2}=0\\ k_{12}v_{1}+\left(-k_{21}-\lambda_{1,2}\right)v_{2}=0. \end{array}\right. \end{array}$$

From (28), we have that:

$$v_{1}=\frac{k_{21}+\lambda_{1,2}}{k_{12}}v_{2},$$

and,

$$v_{2}=\frac{k_{10}+k_{12}+\lambda_{1,2}}{k_{21}}v_{1}.$$

Suppose $v_{2}=1,$ then we have that,

$$v_{1}=\frac{k_{21}+\lambda_{1,2}}{k_{12}},$$

and thus,

$$\underline{v}=\left(\begin{array}{c} v_{1}\\ v_{2} \end{array}\right)=\left(\begin{array}{c} \frac{k_{21}+\lambda_{1,2}}{k_{12}}\\ 1 \end{array}\right).$$

Let $\tau_{1,2}=\frac{K_{21}+\lambda_{1,2}}{K_{12}},$ then we find the corresponding eigenvectors to the eigenvalues to be:

$$\underline{v}=\left(\begin{array}{c} v_{1}\\ v_{2} \end{array}\right)=\left(\begin{array}{c} \tau_{1,2}\\ 1 \end{array}\right).$$

Substituting the value of $\lambda_{1,2}$ from (27), we have:

$$\tau_{1,2}=\frac{-k_{10}-k_{12}+k_{21}\pm \sqrt{\left(k_{10}+k_{12}+k_{21}\right){}^{2}-4k_{10}k_{21}}}{2k_{12}}.$$

We can now write our complementary solution as:

(29) $$q_{c}\left(t\right)=A_{1}e^{\lambda_{1}t}\left(\begin{array}{c} \tau_{1}\\ 1 \end{array}\right)+A_{2}e^{\lambda_{2}t}\left(\begin{array}{c} \tau_{2}\\ 1 \end{array}\right).$$

The general solution to (14) is then of the form $q\left(t\right)=q_{p}+q_{c}$, hence,

(30) $$q\left(t\right)=\left(\begin{array}{c} c(t)\\ p(t) \end{array}\right)=\frac{I_{0}}{k_{10}}\left(\begin{array}{c} 1\\ \frac{k_{12}}{k_{21}} \end{array}\right)+A_{1}e^{\lambda_{1}t}\left(\begin{array}{c} \tau_{1}\\ 1 \end{array}\right)+A_{2}e^{\lambda_{2}t}\left(\begin{array}{c} \tau_{2}\\ 1 \end{array}\right),$$

giving,

(31) $$c\left(t\right)=\frac{I_{0}}{k_{10}}+A_{1}\tau_{1}e^{\lambda_{1}t}+A_{2}\tau_{2}e^{\lambda_{2}t}$$

and,

(32) $$p\left(t\right)=\frac{k_{12}I_{0}}{k_{10}k_{21}}+A_{1}e^{\lambda_{1}t}+A_{2}e^{\lambda_{2}t}.$$

Given the initial condition $\left(\begin{array}{c} c(0)\\ p(0) \end{array}\right)=\left(\begin{array}{c} 0\\ 0 \end{array}\right),$ and employing Equations (31) and (32) we have:

(33) $$\frac{I_{0}}{k_{10}}+A_{1}\tau_{1}+A_{2}\tau_{2}=0,$$

(34) $$\frac{k_{12}I_{0}}{k_{10}k_{21}}+A_{1}+A_{2}=0.$$

With some algebraic manipulation we find the arbitrary constants:

(35) $$A_{1}=\frac{I_{0}}{k_{10}}\left(\frac{k_{12}\tau_{2}-k_{21}}{k_{21}\left(\tau_{1}-\tau_{2}\right)}\right)$$

and,

(36) $$A_{2}=\frac{I_{0}}{k_{10}}\left(\frac{k_{21}-k_{12}\tau_{1}}{k_{21}\left(\tau_{1}-\tau_{2}\right)}\right).$$

Our final solution is then given as:

(37) $$q\left(t\right)=\left(\begin{array}{c} c(t)\\ p(t) \end{array}\right)=\frac{I_{0}}{k_{10}}\left(\begin{array}{c} 1\\ \frac{k_{12}}{k_{21}} \end{array}\right)+\frac{I_{0}}{k_{10}}\left(\frac{k_{12}\tau_{2}-k_{21}}{k_{21}\left(\tau_{1}-\tau_{2}\right)}\right)e^{\lambda_{1}t}\left(\begin{array}{c} \tau_{1}\\ 1 \end{array}\right)+\frac{I_{0}}{k_{10}}\left(\frac{k_{21}-k_{12}\tau_{1}}{k_{21}\left(\tau_{1}-\tau_{2}\right)}\right)e^{\lambda_{2}t}\left(\begin{array}{c} \tau_{2}\\ 1 \end{array}\right).$$

###### 3.2.3. Remarks

As provided in Table 1 the parameter values for $k_{10},k_{12}$ and $k_{21}$ were obtained from [28]. We also consider the case where $k_{12}=0$ as a means of comparison; this case indicates that the transfer rate of the drug from the central to the peripheral compartment is zero.

**Figure 2:**
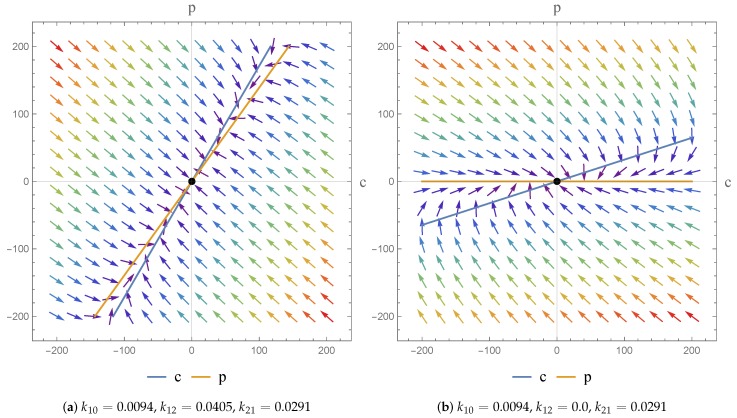
Phase portrait representing c(t) and p(t). The arrows illustrates the trajectories of the system.

Consider the dynamics presented in Figure 2 for the homogeneous case; we find that the transition from $k_{12}>0$ to $k_{12}=0$ does not change the nature of the equilibrium points. In both cases we find that $\lambda_{1}<\lambda_{2}<0$, indicating that we have a node which is defined to be asymptotically stable. This has been checked for the range of parameter values given in Table 1, for Subjects 1–4. This indicates that when the transfer rate of the drug from the central to peripheral compartment is positive, or when there is no transfer, the system maintains the same dynamics.

**Figure 3:**
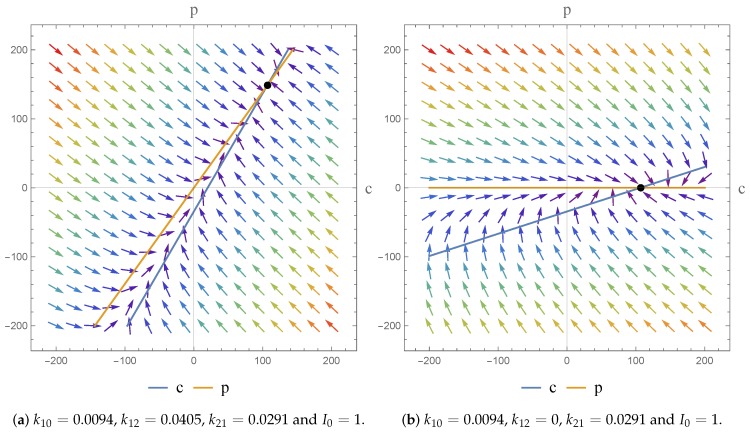
Phase portrait representing c(t) and p(t). The arrows illustrates the trajectories of the system.

Figure 3 shows that for the transition from $k_{12}>0$ to $k_{12}=0$, in the non-homogeneous case, our dynamics once again remain unchanged. We have a nodal sink—defined to be asymptotically stable—at a point where *c* and p>0, such that the concentration in both compartments is positive. In the second instance ($k_{12}=0$) we obtain a point where p=0, i.e., only the concentration in the central compartment is not zero. Thus, we further note that our results have shown that the case where $k_{10}>k_{12}=0$ results in a lower steady-state drug concentration.

**Figure 4:**
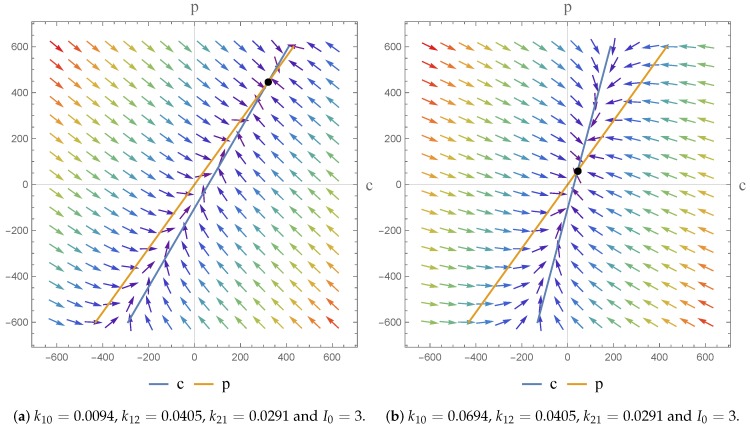
Phase portrait (**a**) shows the steady state concentration is directly proportional to the infusion rate ($I_{0}$). Thus, a higher $I_{0}$ will result in a higher steady-state concentration, and (**b**) shows a higher elimination rate ($k_{10}$) will result in a lower steady-state concentration.

We also note—as indicated in Figure 4a—that the steady state concentration is directly proportional to the infusion rate $I_{0}$. Hence, a higher value of $I_{0}$ will result in a higher steady-state concentration. In Figure 4b we note that a higher elimination rate ($k_{10}$) will result in a lower steady-state concentration.

In terms of an investigation of the impact of $I_{0}$ we note that the drug is given by I.V. infusion at a constant zero-order rate to allow accurate control of the drug concentration and to maintain the level of the drug in the body as constant. This allows us to predict the PK action. For physically meaningful results we can only consider constant values of the dose; at different constant values ($I_{0}$) we obtain a higher infusion rate which results in a higher steady state concentration. We find however, that an increase in $I_{0}$ does not have any effect on the dynamics of the system.

##### 3.3. Nonstandard Finite Difference Scheme

###### 3.3.1. Case 1

Before we discuss the implementation of the NSFD scheme we briefly review the SFD schemes of the system of equations given by (13) which are given as:

(38) $$\begin{array}{ccc} \frac{c_{n+1}-c_{n}}{h} & = & k_{21}p_{n}-k_{12}c_{n}-k_{10}c_{n} \end{array}$$

(39) $$\begin{array}{ccc} \frac{p_{n+1}-p_{n}}{h} & = & k_{12}c_{n}-k_{21}p_{n}. \end{array}$$

Via the SFD method the system may be written in explicit form as:

(40) $$\begin{array}{ccc} c_{n+1} & = & c_{n}+h\left(k_{21}p_{n}-k_{12}c_{n}-k_{10}c_{n}\right) \end{array}$$

(41) $$\begin{array}{ccc} p_{n+1} & = & p_{n}+h\left(k_{12}c_{n}-k_{21}p_{n}\right). \end{array}$$

We now turn to the focus of this section, which is the implementation of the concepts of the NSFD scheme to the linear PK model (13) under discussion [14]. Employing the method discussed in Section 2 we obtain:

(42) $$\begin{array}{ccc} \frac{c_{n+1}-\varphi c_{n}}{\phi } & = & k_{21}p_{n}-k_{12}c_{n}-k_{10}c_{n} \end{array}$$

(43) $$\begin{array}{ccc} \frac{p_{n+1}-\varphi p_{n}}{\phi } & = & k_{12}c_{n}-k_{21}p_{n}. \end{array}$$

where,

$$\phi =\frac{e^{\lambda_{1}h}-e^{\lambda_{2}h}}{\lambda_{1}-\lambda_{2}}\;\;\mathrm{and}\;\;\varphi =\frac{\lambda_{1}e^{\lambda_{2}h}-\lambda_{2}e^{\lambda_{1}h}}{\lambda_{1}-\lambda_{2}},$$

and $\lambda_{1}$ and $\lambda_{2}$ are given as per Equation (17).

###### 3.3.2. Case 2:

As before, we present the SFD schemes of the system of equations given by (14) before discussing the relevant NSFD scheme. These are given as:

(44) $$\begin{array}{ccc} \frac{c_{n+1}-c_{n}}{h} & = & k_{21}p_{n}-k_{12}c_{n}-k_{10}c_{n}+I_{0} \end{array}$$

(45) $$\begin{array}{ccc} \frac{p_{n+1}-p_{n}}{h} & = & k_{12}c_{n}-k_{21}p_{n}. \end{array}$$

This system may be written in explicit form as:

(46) $$\begin{array}{ccc} c_{n+1} & = & c_{n}+h\left(k_{21}p_{n}-k_{12}c_{n}-k_{10}c_{n}+I_{0}\right) \end{array}$$

(47) $$\begin{array}{ccc} p_{n+1} & = & p_{n}+h\left(k_{12}c_{n}-k_{21}p_{n}\right). \end{array}$$

We now turn to the NSFD implementation as a means of solution. In order to do so we start by considering the system of equations given by (14) which, under the requirement:

(48) $$k_{10}k_{21}\neq 0,$$

gives the general solutions:

(49) $$\begin{array}{c} c\left(t\right)=\frac{1}{\lambda_{1}-\lambda_{2}}\left(\left(k_{10}k_{21}-\lambda_{2}\right)c_{0}+k_{21}p_{0}+\frac{I_{0}\left(\lambda_{2}-k_{10}k_{21}-k_{12}\right)}{k_{10}}\right)e^{\lambda_{1}\left(t-t_{0}\right)}+\\ \frac{1}{\lambda_{1}-\lambda_{2}}\left(\left(\lambda_{1}-k_{10}k_{21}\right)c_{0}+k_{21}p_{0}+\frac{I_{0}\left(k_{10}k_{21}-\lambda_{2}+k_{12}\right)}{k_{10}}\right)e^{\lambda_{2}\left(t-t_{0}\right)}+\frac{I_{0}}{k_{10}}, \end{array}$$

(50) $$\begin{array}{c} p\left(t\right)=\frac{\lambda_{1}+k_{10}+k_{12}}{k_{21}\left(\lambda_{1}-\lambda_{2}\right)}\left(\left(k_{10}k_{21}-\lambda_{2}\right)c_{0}+k_{21}p_{0}+\frac{I_{0}\left(\lambda_{2}-k_{10}k_{21}-k_{12}\right)}{k_{10}}\right)e^{\lambda_{1}\left(t-t_{0}\right)}+\\ \frac{\lambda_{2}+k_{10}+k_{12}}{k_{21}\left(\lambda_{1}-\lambda_{2}\right)}\left(\left(\lambda_{1}-k_{10}k_{21}\right)c_{0}+k_{21}p_{0}+\frac{I_{0}\left(k_{10}k_{21}-\lambda_{2}+k_{12}\right)}{k_{10}}\right)e^{\lambda_{2}\left(t-t_{0}\right)}+\frac{k_{12}I_{0}}{k_{10}k_{21}}, \end{array}$$

where $\lambda_{12}$ is given in Equation (27).

The NSFD scheme of Equation (14) is obtained by making the following transformation in Equations (49) and (50):

(51) $$\begin{array}{c} \left\{\begin{array}{c} t_{0}\to t_{k}=hk,\\ t\to t_{k+1}=h\left(k+1\right),\\ u_{0}\to u_{k},\\ u\left(t\right)\to u_{k+1},\\ w_{0}\to w_{k},\\ w\left(t\right)\to w_{k+1}, \end{array}\right. \end{array}$$

giving the following results:

(52) $$\begin{array}{c} c_{k+1}=\frac{1}{\lambda_{1}-\lambda_{2}}\left(\left(k_{10}k_{21}-\lambda_{2}\right)c_{k}+k_{21}p_{k}+\frac{I_{0}\left(\lambda_{2}-k_{10}k_{21}-k_{12}\right)}{k_{10}}\right)\\ e^{\lambda_{1}h}+\frac{1}{\lambda_{1}-\lambda_{2}}\left(\left(\lambda_{1}-k_{10}k_{21}\right)c_{k}+k_{21}p_{k}+\frac{I_{0}\left(k_{10}k_{21}-\lambda_{2}+k_{12}\right)}{k_{10}}\right)e^{\lambda_{2}h}+\frac{I_{0}}{k_{10}}, \end{array}$$

(53) $$\begin{array}{c} p_{k+1}=\frac{\lambda_{1}+k_{10}+k_{12}}{k_{21}\left(\lambda_{1}-\lambda_{2}\right)}\left(\left(k_{10}k_{21}-\lambda_{2}\right)c_{k}+k_{21}p_{k}+\frac{I_{0}\left(\lambda_{2}-k_{10}k_{21}-k_{12}\right)}{k_{10}}\right)e^{\lambda_{1}h}+\\ \frac{\lambda_{2}+k_{10}+k_{12}}{k_{21}\left(\lambda_{1}-\lambda_{2}\right)}\left(\left(\lambda_{1}-k_{10}k_{21}\right)c_{k}+k_{21}p_{k}+\frac{I_{0}\left(k_{10}k_{21}-\lambda_{2}+k_{12}\right)}{k_{10}}\right)e^{\lambda_{2}h}+\frac{k_{12}I_{0}}{k_{10}k_{21}}. \end{array}$$

## 4. Discussion

Due to its simplicity, the compartment model often serves as a “first model” that requires further refinement in order to describe the physiologic and drug distribution processes in the body accurately. While this may be the case, non-compartment models have gained popularity in the literature due to their ability to easily relate experimental data to mathematical models via parameter fitting. Foste [31] points out that upon comparing non-compartment with compartment models, it is not a question of declaring one method better than the other. It is a question of (1) what information is desired from the data; and (2) what is the most appropriate method to obtain this information. Known limitations of non-compartment models include the fact that they do not allow for meta-analysis and the deeper insight that the physiological-based PK models allow for [32]. Another important limitation of non-compartment PK analysis is that it lacks the ability to predict PK profiles when there are alterations in a dosing regimen, which compartment PK methods are capable of [33]. Furthermore, compartmental approaches allow for some level of “physiological” interpretation of what the body does to the drug (i.e., consistency with physiologically reasonable pathways of drug elimination can be maintained). Shargel et al. [8] note that, in comparison to non-compartment models, compartment models are particularly useful when little information is known about the tissues of the compartment(s). In this study non-compartment models are not of interest, not due to their limitations but because non-compartment analysis is based on algebraic equations, whereas compartment models are based on linear or nonlinear differential equations. We instead investigate the use of a method which is designed for the effective solution of differential equations, and as such turn our attention away from non-compartment models.

In this study two-compartment models (the I.V. bolus injection and I.V. infusion models) are considered for simulations. The methods compared are the SFD method, and the in-built function ODE45 in MATLAB, with the focus of the study being the NSFD method. All the simulations were performed by using MATLAB. The absolute error is presented using the formula:

$$E_{\mathrm{Absolute}}=\left|X_{\mathrm{Analytical}\;\mathrm{solution}}-X_{\mathrm{Numerical}\;\mathrm{solution}}\right|.$$

The first column, in each table presented, is the number of nodes chosen, the second column is the corresponding step size, the third column is the absolute error between the exact solution and the SFD scheme, the fourth column is the absolute error between the exact solution and ODE45 in MATLAB, and the last column gives the absolute error between the exact solution and the NSFD scheme.

Once again, we remind the reader of the values provided in Table 1 for the parameters: $k_{10},k_{12}$ and $k_{21}$ [28]. These were derived from a two-compartment open model analysis of serum of sisomicin after I.V. administration.

### 4.1. Case 1: Simulation Results

**Figure 5:**
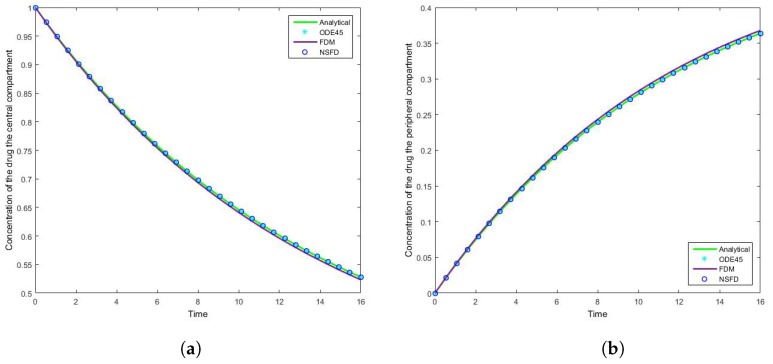
The concentration of the drug for Case 1 where h=0.53333 in the (**a**) central compartment, and (**b**) peripheral compartment.

**Figure 6:**
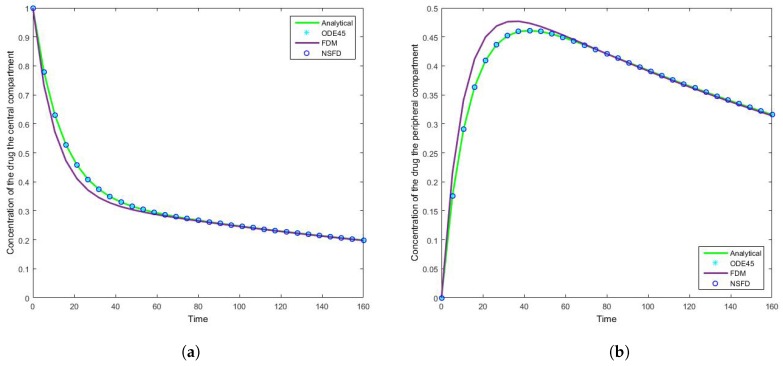
The concentration of the drug for Case 1 where h=5.3333 in the (**a**) central compartment, and (**b**) peripheral compartment for a large time interval.

**Figure 7:**
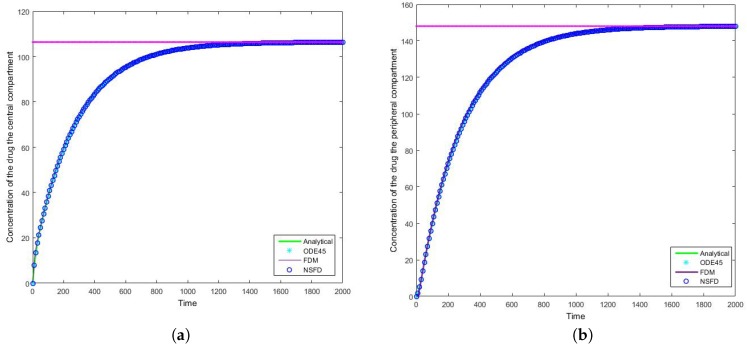
The concentration of the drug for Case 2 where h=10 in the (**a**) central compartment, and (**b**) peripheral compartment. At the steady state, the rate of drug infusion ($I_{0}$) is equal to the elimination rate ($k_{10}$).

The results for this case clearly indicate the degree to which the NSFD method outperforms the SFD method, and in fact the in-built function as well—see Figure 5. For each step size employed the absolute error calculated is in fact zero, as per see Table 2 and Table 3, given that the NSFD method produces “exact” solutions. More importantly, we see from Figure 6 and Figure 7 that for large step sizes the NSFD method performs exceptionally well, matching the analytical solution for the entire time profile, while the SFD method is not able to do so.

The SFD method deviates from the exact solution the moment the solution changes gradient—see Figure 7. This is a known problem for methods of this nature; we find that as expected the NSFD method does not suffer from this weakness.

### 4.2. Case 2: Simulation Results

**Figure 8:**
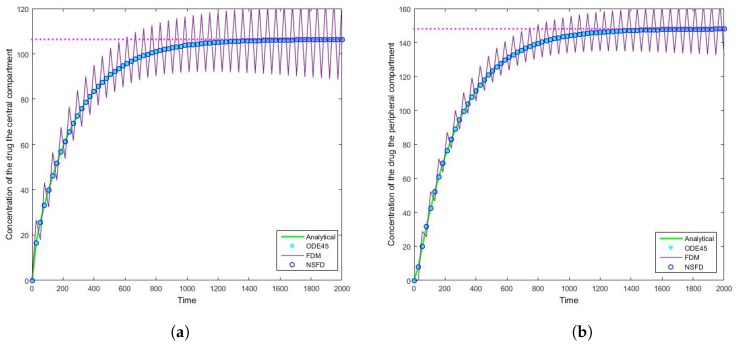
The concentration of the drug for Case 2 where h=26.667 in the (**a**) central compartment, and (**b**) peripheral compartment.

Once more we find that for each step size employed the NSFD method outperforms those methods it is compared to—see Table 4 and Table 5. In this case in particular, the structuring of the NSFD scheme is shown to be quite complex. The NSFD scheme in this case was obtained via an initial structuring of the exact solution of the problem and making the substitution provided in Equation (51). We note from Figure 8 that the schemes reach the steady state as required. More importantly we observe the oscillations which occur for increasing step sizes *h*. It must be noted that these step sizes are realistic given that t∈[0,2000]; as such in a range of [0,1] the value of h=10 represents Δt=0.005, whereas h=26.667 is represented by Δt=0.013. We can easily observe here the degree to which the NSFD method is able to outperform the other two numerical methods employed. The SFD scheme does not match the real dynamics of the system for higher step sizes; we observe oscillations of the SFD method for large *h*. As the step size increases there is complete blowup of the SFD scheme and the error in ODE45 increases.

In terms of an investigation of the impact of $I_{0}$ we note that the drug is given by I.V. infusion at a constant zero-order rate to allow accurate control of the drug concentration and to maintain the level of the drug in the body as constant. This allows us predict PK action. For physically meaningful results we can only consider constant values of the dose; at different constant values ($I_{0}$) we obtain a higher infusion rate which results in a higher steady state concentration.

## 5. Conclusions

In this article we structured and applied a NSFD numerical scheme to two systems of equations which are both PK models. The first model is an I.V. bolus injection two-compartment model while the second model is an I.V. infusion two-compartment model. The systems are homogeneous and non-homogeneous systems of differential equations, respectively. We present some absolute errors associated with this method upon comparison to the relevant analytical solution, the SFD method and the in-built function ODE45 in MATLAB. The NSFD results indicates superior performance over the SFD method and the in-built MATLAB function, confirming both its stability and robustness.

We conclude that the developed nonstandard schemes preserve the significant properties of their continuous analogues and consequently give reliable numerical results. It was found that the NSFD schemes were stable for large step sizes and display qualitatively accurate results, allowing one to assess and predict the long time behaviour of the drug in the system. Given that the NSFD method produces “exact” numerical schemes, we may conclude that they are well-suited for the solution of pharmacokinetic models.

The flexibility afforded by the NSFD scheme, in terms of its construction, means that the scheme secures consistency with the continuous pharmacokinetic models arising from compartmental or physiological models with respect to the different dynamical characteristics of the systems [17]. As a consequence, we were able to investigate the dynamics of the models and the impact of the parameter value choices employed. We observe asymptotically stable dynamics in each case and note the impact of parameter value choices on the equilibrium states obtained.

It now remains to extend this method to more complex systems of equations, such as non-linear and/or multi-compartment PK models.

## Figures and Tables

**Table 1:** Values for sisomicin kinetic parameters derived from two-compartment open model analysis of serum data after intravenous (I.V.) administration. These values are visualized in Figure 1 and are obtained from the work by Péchère et al. [28].

Parameter	Unit	Value: Subject 1	Value: Subject 2	Value: Subject 3	Value: Subject 4
$k_{10}$	$min^{-1}$	0.00940	0.01110	0.01030	0.01520
$k_{12}$	$min^{-1}$	0.04050	0.02504	0.02750	0.04120
$k_{21}$	$min^{-1}$	0.02910	0.02230	0.02830	0.02410
*D*	mg/min	1.00000	1.00000	1.00000	1.00000

**Table 2:** The absolute error results for the system of equations given by (13) for c(t) with parameter values $k_{10}=0.0094$, $k_{12}=0.0405$, and $k_{21}=0.0291$.

Absolute Error for c(t)				
**N**	**h**	**Error in SFD**	**Error in ODE45**	**Error in NSFD**
2	10.00000	0.144913386	0.000000010	0.000000000
4	5.00000	0.053199386	0.000000010	0.000000000
8	2.50000	0.024396686	0.000000010	0.000000000
16	1.25000	0.011669111	0.000000022	0.000000000
32	0.62500	0.005719559	0.000000028	0.000000000
64	0.31250	0.002831162	0.000000028	0.000000000
128	0.15625	0.001408639	0.000000028	0.000000000
256	0.07812	0.000702591	0.000000028	0.000000000
512	0.03906	0.000350865	0.000000028	0.000000000
1024	0.01953	0.000175326	0.000000029	0.000000000

**Table 3:** The absolute error results for the system of equations given by (13) for *p* with parameter values $k_{10}=0.0094$, $k_{12}=0.0405$, and $k_{21}=0.0291$.

Absolute Error for p(t)				
**N**	**h**	**Error in SFD**	**Error in ODE45**	**Error in NSFD**
2	10.00000	0.126270717	0.000000009	0.000000000
4	5.00000	0.046283217	0.000000009	0.000000000
8	2.50000	0.021180830	0.000000009	0.000000000
16	1.25000	0.010127446	0.000000019	0.000000000
32	0.62500	0.004961080	0.000000025	0.000000000
64	0.31250	0.002455508	0.000000025	0.000000000
128	0.15625	0.001221609	0.000000025	0.000000000
256	0.07812	0.000609284	0.000000025	0.000000000
512	0.03906	0.000304265	0.000000025	0.000000000
1024	0.01953	0.000152038	0.000000026	0.000000000

**Table 4:** The absolute error results for the system of equations given by (14) for c(t) with parameter values $k_{10}=0.0094$, $k_{12}=0.0405$, $k_{21}=0.0291$ and $I_{0}=1$.

Absolute Error for c(t)				
**N**	**h**	**Error in SFD**	**Error in ODE45**	**Error in NSFD**
2	10.00000	1.98326261	0.00000008	0.00000000
4	5.00000	0.73839463	0.00000010	0.00000000
8	2.50000	0.34211236	0.00000012	0.00000000
16	1.25000	0.16473051	0.00000018	0.00000000
32	0.62500	0.08083009	0.00000021	0.00000000
64	0.31250	0.04005593	0.00000019	0.00000000
128	0.15625	0.01993844	0.00000022	0.00000000
256	0.07812	0.00994725	0.00000023	0.00000000
512	0.03906	0.00496814	0.00000022	0.00000000
1024	0.01953	0.00248270	0.00000023	0.00000000

**Table 5:** The absolute error results for the system of equations given by (14) for p(t) with parameter values $k_{10}=0.0094$, $k_{12}=0.0405$, $k_{21}=0.0291$ and $I_{0}=1$.

Absolute Error for p(t)				
**N**	**h**	**Error in SFD**	**Error in ODE45**	**Error in NSFD**
2	10.00000	1.57898904	0.00000007	0.00000000
4	5.00000	0.56648904	0.00000009	0.00000000
8	2.50000	0.25172781	0.00000011	0.00000000
16	1.25000	0.12005326	0.00000016	0.00000000
32	0.62500	0.05860324	0.00000018	0.00000000
64	0.31250	0.02895478	0.00000017	0.00000000
128	0.15625	0.01439233	0.00000020	0.00000000
256	0.07812	0.00717515	0.00000021	0.00000000
512	0.03906	0.00358234	0.00000019	0.00000000
1024	0.01953	0.00178986	0.00000020	0.00000000

## Abbreviations

The following abbreviations are used in this manuscript:

PK	pharmacokinetic
PD	pharmacodynamic
I.V.	intravenous
NSFD	nonstandard finite difference
SFD	standard finite difference

## References

[1] Buchanan A. (1847). Physiologic effects of the inhalation of ether. Lond. Med. Gaz..

[2] Michealis L., Menten M.L. (1913). Die Kinetik der Invertinwirking. Biochem. Z..

[3] Widmark E., Tandberg J. (1924). Uber die bedingungen f’tir die Akkumulation Indifferenter Narkoliken Theoretische Bereckerunger. Biochem. Z..

[4] Teorell T. (1937). Kinetics of distribution of substances administered to the body. I. The extravascular modes of administration. Arch. Int. Pharmacodyn. Ther..

[5] Holford N.H.G., Sheiner L.B. (1982). Kinetics of pharmacologic response. Pharmacol. Ther..

[6] Huang S.-M., Abernethy D.R., Wang Y., Zhao P., Zineh I. (2013). The utility of modelling and simulation in drug development and regulatory review. J. Pharm. Sci..

[7] Bonate P.L. (2011). Pharmacokinetic-Pharmacodynamic Modelling and Simulation.

[8] Shargel L., Yu A., Wu-Pong S. (2012). Introduction to Biopharmaceutics and Pharmacokinetics. Applied Biopharmaceutics & Pharmacokinetics.

[9] Shargel L., Yu A., Wu-Pong S. (2012). Multicompartment Models: Intravenous Bolus Administration. Applied Biopharmaceutics & Pharmacokinetics.

[10] Shargel L., Yu A., Wu-Pong S. (2012). Physiologic Pharmacokinetic Models, Mean Residence Time, and Statistical Moment Theory. Applied Biopharmaceutics & Pharmacokinetics.

[11] Beňová M., Gombárska D., Dobrcký B. (2013). Using Euler’s and Taylor’s expansion method for solution of non-linear differential equation system in pharmacokinetics. Electr. Rev..

[12] Mickens R.E. (2005). Dynamic consistency: A fundamental principle for constructing nonstandard finite difference schemes for differential equations. J. Differ. Equ. Appl..

[13] Mickens R.E. (2000). Application of Nonstandard Finite Difference Schemes.

[14] Mickens R.E. (2002). Nonstandard finite difference schemes for differential equations. J. Differ. Equ. Appl..

[15] Mickens R.E. (2003). A nonstandard finite difference scheme for the diffusionless Burgers equation with logistic reaction. Math. Comput. Simul..

[16] Mickens R.E. (2005). A numerical integration technique for conservative oscillators combining nonstandard finite-difference methods with a Hamilton’s principle. J. Sound Vib..

[17] Liao C., Ding X. (2013). Nonstandard finite difference variational integrators for nonlinear Schrödinger equation with variable coefficients. Adv. Differ. Equ..

[18] Arenas A.J., González-Parra G., Chen-Charpentier B.M. (2010). A nonstandard numerical scheme of predictor-corrector type for epidemic models. Comput. Math. Appl..

[19] González-Parra G., Arenas A.J., Chen-Charpentier B.M. (2010). Combination of nonstandard schemes and Richardson’s extrapolation to improve the numerical solution of population models. Math. Comput. Model..

[20] Jordan P.M. (2003). A nonstandard finite difference scheme for nonlinear heat transfer in a thin finite rod. J. Differ. Equ. Appl..

[21] Malek A. (2011). Applications of nonstandard finite difference methods to nonlinear heat transfer problems. Heat Transfer—Mathematical Modelling, Numerical Methods and Information Technology.

[22] Introduction to Non-Standard Finite-Difference Modelling. https://www.crewes.org/ForOurSponsors/ResearchReports/2006/2006-46.pdf.

[23] Mickens R.E. (1988). Lie methods in mathematical modelling: Difference equation models of differential equation. Math. Comput. Model..

[24] Mickens R.E. (2003). Exact solutions to a finite-difference model of a nonlinear reaction-advection equation: Implications for numerical analysis. J. Differ. Equ. Appl..

[25] Mickens R.E. (1994). Nonstandard Finite Difference Models of Differential Equations.

[26] Anguelov R., Lubuma J.M.-S. (2003). Nonstandard finite difference method by nonlocal approximation. Math. Comput. Simul..

[27] Gurski K.F. (2013). A simple construction of nonstandard finite-difference schemes for small nonlinear systems applied to SIR models. Comput. Math. Appl..

[28] Péchère J.-C., Péchère M.-M., Dugal R. (1976). Clinical Pharmacokinetics of Sisomicin: Two-Compartment Model Analysis of Serum Data after I.V. and I.M. Administration. Eur. J. Clin. Pharmacol..

[29] Widder D.V. (1966). The Laplace Transform.

[30] Koch G. (2012). Modeling of Pharmacokinetics and Pharmacodynamics with Application to Cancer and Arthritis. Ph.D. Thesis.

[31] Foster D.M., Atkinson A.J., Abernethy D.R., Daniels C.E., Dedrick R.L., Markey S.P. (2014). Noncompartmental versus Compartmental Approaches to Pharmacokinetic Analysis. Principles of Clinical Pharmacology.

[32] Wang Z., Kim S., Quinney S.K., Zhou J., Li L. (2010). Non-compartment model to compartment model pharmacokinetics transformation meta-analysis–a multivariate nonlinear mixed model. BMC Syst. Biol..

[33] Jang G.R., Harris R.Z., Lau D.T. (2001). Pharmacokinetics and its role in small molecule drug discovery research. Med. Res. Rev..

